# 

**Published:** 1900-02

**Authors:** 


### Communicated.

---

### THE INTERNATIONAL TOOTH CROWN COMPANY VS. KYLE.

---

### INTERPRETATION OF THE DECISION.

---

Since the recent decision in the International Tooth Crown Company's suit, we have received many letters and inquiries from dentists, in various parts of the country, asking for information respecting it.

These inquiries plainly indicate that there is a very general misunderstanding among the dentists as to the nature of the decision.

The idea appears to be prevalent among the profession that they, as individual dentists, are liable for work which they are now doing, and that the decision covers crowns of all sorts, including porcelain, as well as solid gold or cap crowns, whether used singly or in bridges.

As this wide-spread misconception affects the sale of certain articles made and sold by this Company, we have thought it proper to make a statement of facts. We therefore publish the following opinion of our counsel, Messrs. Fraley & Paul:

No. 929 Chestnut Street, Philadelphia.

THE S. S. WHITE DENTAL MANUFACTURING COMPANY.

*Gentlemen.*—As requested by you, we have carefully examined the recent decision of the United States Circuit Court for the Southern District of New York, in the suit of the International Tooth Crown Company against James Orr Kyle. The patent sued upon is that of James E. Low, No. 238,940, of March 15th, 1881, the claims of which are as follows:

1. "The herein described method of inserting and supporting artificial teeth, which consists in attaching said artificial teeth to continuous bands fitted and cemented to the adjoining permanent teeth, whereby said artificial teeth are supported by said permanent teeth without dependence upon the gum beneath."

2. "An artificial tooth cut away at the back, so as not to present any contact with the gum, except along its front lower edge, and supported by rigid attachment to one or more adjoining permanent teeth, substantially as and for the purpose set forth."

Without attempting to discuss the patent at length, or to define what possible construction could be put upon the claims, we point out that it only covers dental *bridge-work*, and not a single crown, of themselves.

We think it proper to call attention to this point, because a person hastily reading the second claim might possibly think that it covered an artificial tooth cut away at the back, irrespective of its attachment to adjoining natural teeth. Such, however, is not the case, and it is perfectly clear that the Logan crowns and the Evans crowns, sold by your Company, are not in the least affected by the decision.

The patent expired on the 15th of March, 1898, and consequently no one can be held liable thereunder for bridge-work made since that date, nor can any injunction hereafter issue to restrain a dentist from using the invention set forth in the patent, as it is now public property. His utmost liability would be for bridge-work done during a period beginning six years before the day the suit was brought, and ending on the 15th of March, 1898. This can now only be recovered by a suit before a jury, as distinguished from the somewhat lengthy and expensive procedure in equity.

We have made this brief statement as to the liability of dentists for past use of the invention, as a matter of general interest, but so far as your own position is concerned two things are perfectly clear. *First*, that the Logan and other porcelain crowns, and the gold cap crowns, sold by you, are not of themselves, and never were, within the claims of the patent, since the latter only covered bridge-work; and, *Second*, that at the present time any one is free to use artificial teeth of various kinds for bridge-work, without liability under the Low patent above referred to.

FRALEY & PAUL.

December 12th, 1899.

THE S. S. WHITE DENTAL MANUFACTURING COMPANY.
— *The Dental Cosmos.*

---

### INTERNATIONAL DENTAL CONGRESS.—REPORT OF TRANSPORTATION COMMITTEE.

The Sub. Committee on Transportation has completed arrangements with the well known tourist firm of Thomas Cook & Sons, 251 Broadway, New York, so that dentists who expect to attend the Congress to be held in Paris, commencing August 8th, 1900, may secure for themselves and family's, steamship and railroad tickets and hotel accommodations at the minimum of expense and trouble.

In making these arrangements the committee has taken into consideration that while some of the delegates may wish to secure only transportation from New York to Paris and back to New York, many delegates will wish to visit other parts of Europe during the summer and they have planned the following tours to assist such in the selection of a trip that the time at their disposal and their means will suggest.

### TOUR I.

a. From New York by Red Star Line Steamer "Friesland" on July 18th, for Antwerp, thence rail via Brussels to Paris returning same way to New Nork. First-class passage providing berth at minimum rate for two berthed room, $157.85.

If traveling second-class from Antwerp to Paris and return fare would be $4.65 less.

By traveling on steamers "Kensington" or "Southwark" of same line, fare would be reduced.

b. Via Cherburg (North German Lloyd Service).

From New York by North German Lloyd Steamers "Barbarosa" and "Friederich de Grosse," sailing July 12th, and 19th, respectfully, for Cherberg, thence rail to Paris and return same way, (twin screw service only). First-class passage, providing berth in room for two persons, (minimum rate), $177.00.

c. Via Cherburg, (Hamburg American Line Service).

From New York by Hamburg American Line Steamers "Pennsylvania" and "Pretoria," sailing July 14th and 21st, respectively to Cherburg, rail to Paris and return via Boulogne-sur-mer and Hamburg American Steamer, (twin screw service), to New York. First-class passage providing minimum fare for berth in room for two persons only, $184.25.

Lower fares can be obtained if occupying berth in room with two or three other occupants.

d. Via Boulogne-sur-mur (Holland American Line).

From New York by twin screw steamers "Potsdam," "Statendam" and "Rotterdam," sailing July 7th, 14th and 28th, respectively, to Boulogne-sur-mur, thence rail to Paris and return same way to New York. First-class passage providing minimum fare for berth in room for two passengers, $163.00.

If traveling second-class from Boulogne to Paris and return, fare would be $3.80 less.

Lower fares can be made by leaving on steamer "Sparndam" July 19th.

Tickets can also be arranged via Southampton or Liverpool at proportionate fares.

### TOUR 2.

To provide hotel accommodation in Paris for two weeks, (14 days and 13 nights) at Grand Hotel du Trocadero, carriage drives for three days, including excursion to St. Cloud and Versailles, 20 tickets of admission to exposition and transfer to and from railway station to hotel, $65.00.

TOUR 3.

One week's tour to Switzerland from Paris, visiting Lucerne, Interlaken, Thun, Berne, Lausanne, Lake Leman, Geneva, including hotel accommodation, sight seeing, etc., second-class R. R., $50.00.

TOUR 4.

One week's tour from Paris to Mayence, thence steamer on Rhine to Cologne, rail to Amsterdam, The Hague, Rotterdam, Antwerp, Brussels, Harwich, London, including second-class railway travel, first-class on steamers, hotel coupons, (3 meals per day, with lodging, $42.50.

Those traveling via Cherburg can return by steamers of same line from Southampton and so make short tour from continent through England in connection.

There is a U. S. revenue tax of $5.00 upon each ticket regardless of the number of passengers in whose name it may be made out.

Should any one wish to make a longer tour than any of the foregoing, or one with a different route, Mess. Cook & Sons have such a large variety of tours already planned that there need be no difficulty in making a selection to suit the taste, means or the time at the disposal of any one.

The war in South Africa has caused the withdrawal of many of the English steamships. Passenger accommodations across the atlantic will be less than usual this summer, while the Paris exposition is attracting great numbers so that the committee wish to impress upon delegates the great importance of securing their steamship accommodations at once.

Address all communications regarding steamships, railroads, hotels, etc., to Mess. Thomas Cook & Sons, No. 251 Broadway, New York.

A. W. HARLAN,
W. E. GRISWOLD,
W. W. WALKER,
WILLIAM JARVIE, Chairman.
Transportation Committee.

### KENTUCKY STATE DENTAL ASSOCIATION.

LOUISVILLE, KY., February 2, 1900.

DR. RICHARD GRADY,

DEAR DOCTOR :—I wish to call your attention to the change of date of the meeting of the Kentucky State Dental Association. On account of change of meeting of Confederate Association, and for the purpose of getting railroad rates, we too have changed our date to May 29, 30, 31. We have some thirty papers promised for the meeting and nearly as many clinics and we will still add others to the list. Please be kind enough to announce this change of date and give us as much other notice as you deem wise. We have men on the programme from about twelve states. Don't fail to come and meet with us and we promise you a fine time.

Yours fraternally,

F. J. GARDNER, D. D. S.,

Secretary.

---

The Board of Dental Examiners of the State of Pennsylvania will conduct examinations simultaneously in Philadelphia and Pittsburg, May 8, 9, and 10, and in Philadelphia June 19, 20 and 21.

Application for examination must be made to the Hon. James W. Latla, Secretary of the Dental Council, Harrisburg, Pa.

G. W. KLUMP, Sec'y,

Williamsport, Pa.

### To Readers and Correspondents.

Communications intended for publication, and exchange journals and papers, should be addressed to the Editor of The American Journal of Dental Science, No. 845 N. Eutaw St. Baltimore, Md. All letters relating to the business of the Journal, or containing cash remittances, to be directed to Snowden & Cowman Mfg. Co., 9 W. Fayette Street, Baltimore, Md. Articles for publication should be received by the Editor at least two weeks previous to the first of the month on which the number in which it is designed for them to appear is issued.

☞ The American Journal of Dental Science will contain fifty pages of reading matter in each number, making a volume of about Six Hundred pages,

EXCLUSIVE OF ADVERTISEMENTS.

